# Unexpected genomic, biosynthetic and species diversity of *Streptomyces* bacteria from bats in Arizona and New Mexico, USA

**DOI:** 10.1186/s12864-021-07546-w

**Published:** 2021-04-07

**Authors:** Cooper J. Park, Nicole A. Caimi, Debbie C. Buecher, Ernest W. Valdez, Diana E. Northup, Cheryl P. Andam

**Affiliations:** 1 Department of Molecular, Cellular and Biomedical Sciences, University of New Hampshire, Durham, NH USA; 2 Department of Biology, University of New Mexico, Albuquerque, NM USA; 3 Buecher Biological Consulting, Tucson, AZ USA; 4 U.S. Geological Survey, Fort Collins Science Center, Fort Collins, CO USA; 5 Department of Biological Sciences, University at Albany, State University of New York, Albany, NY USA

**Keywords:** *Streptomyces*, Bats, Genome, Average nucleotide identity, Biosynthetic gene clusters

## Abstract

**Background:**

Antibiotic-producing *Streptomyces* bacteria are ubiquitous in nature, yet most studies of its diversity have focused on free-living strains inhabiting diverse soil environments and those in symbiotic relationship with invertebrates.

**Results:**

We studied the draft genomes of 73 *Streptomyces* isolates sampled from the skin (wing and tail membranes) and fur surfaces of bats collected in Arizona and New Mexico. We uncovered large genomic variation and biosynthetic potential, even among closely related strains. The isolates, which were initially identified as three distinct species based on sequence variation in the 16S rRNA locus, could be distinguished as 41 different species based on genome-wide average nucleotide identity. Of the 32 biosynthetic gene cluster (BGC) classes detected, non-ribosomal peptide synthetases, siderophores, and terpenes were present in all genomes. On average, *Streptomyces* genomes carried 14 distinct classes of BGCs (range = 9–20). Results also revealed large inter- and intra-species variation in gene content (single nucleotide polymorphisms, accessory genes and singletons) and BGCs, further contributing to the overall genetic diversity present in bat-associated *Streptomyces*. Finally, we show that genome-wide recombination has partly contributed to the large genomic variation among strains of the same species.

**Conclusions:**

Our study provides an initial genomic assessment of bat-associated *Streptomyces* that will be critical to prioritizing those strains with the greatest ability to produce novel antibiotics. It also highlights the need to recognize within-species variation as an important factor in genetic manipulation studies, diversity estimates and drug discovery efforts in *Streptomyces*.

**Supplementary Information:**

The online version contains supplementary material available at 10.1186/s12864-021-07546-w.

## Background

Many of the drugs used against infectious diseases and other medical disorders have been historically derived from molecules synthesized by environmental microbes, with the most notable belonging to the genus *Streptomyces* (phylum Actinobacteria) [1–3]. The genotypic and phenotypic diversity of *Streptomyces* is remarkably enormous. The current, estimated number of known *Streptomyces* species is approximately 650 [4], thus making it one of the largest genera in the bacterial domain. *Streptomyces* are ubiquitous in the environment. They are often found in soil and decaying vegetation [5, 6], as well as in extreme environments such as polar regions [7, 8], deserts [9], hypersaline sites [10] and the deep sea [11]. Some species form a symbiotic relationship with invertebrates [12], many of which use *Streptomyces*-produced antibiotics to protect themselves against infection [12, 13]. For example, beewolf digger wasps cultivate symbiotic *Streptomyces* species that produce a cocktail of multiple antibiotics for protection against infections [14]. Wasps then deposit a combination prophylaxis of nine different antibiotics into the larval cocoon, a defensive strategy similar to the combination treatment used in human medicine [14]. This results in higher efficacy against a broader spectrum of pathogens and reduces the likelihood of a pathogen developing resistance [14].

The increasing public health burden caused by multidrug resistance and the continuing need to find new treatments against communicable (infectious) and non-communicable (chronic) diseases suggests that the search for bioactive compounds with novel mechanisms of action or with new cellular targets is greater than ever. Unexplored or rarely visited sites, such as caves that have unique physical and chemical characteristics (e.g., high humidity, low light, limited nutrients), represent a fertile source of antibiotic-producing bacteria for potential use in drug discovery efforts. For example, a genetically diverse assembly of *Streptomyces* have been identified in various volcanic, limestone and other calcareous caves, including those found on cave walls and in guano [15–18]. When tested against a variety of fungal and bacterial pathogens, some of these *Streptomyces* exhibited antagonistic activity, thus providing a rich reservoir of pharmaceutically relevant bioactive molecules. Cave-dwelling animals such as bats have also been shown to harbor diverse *Streptomyces* bacteria, many of which have the ability to inhibit the invasive fungus *Pseudogymnoascus destructans* [19]. This fungus is the causal agent of white-nose syndrome that affects hibernating bats and has resulted in reduced bat populations in North America [20].

The ability of *Streptomyces* to successfully inhabit many underexplored or overlooked environments suggests that many novel bioactive compounds remain to be discovered. In this study, we used genomic approaches to explore the diversity of 73 *Streptomyces* isolates collected from multiple species of bats inhabiting caves in Arizona and New Mexico [19]. Results indicate a remarkably diverse array of *Streptomyces* species from bats, based on genome-wide average nucleotide identity (ANI) [21]. We also report inter- and intra-species variation in gene content and biosynthetic gene clusters, which further expands the metabolic potential of these bacteria. Our findings provide important insights on bats and caves as unique but poorly studied environmental sources of antibiotic-producing *Streptomyces*. This knowledge will be critical to addressing the urgent need to discover commercial antibiotics with novel cellular targets or novel molecular activity to inhibit pathogens that threaten the health of humans, bats and other animals.

## Results

### Discordance in species boundaries between 16S rRNA gene and genome-wide nucleotide identity

We obtained whole genome sequences of 73 *Streptomyces* isolates that were sampled from the skin and fur surfaces of healthy bats (i.e., free of white-nose syndrome) that were collected from multiple caves in Arizona and New Mexico, USA (Additional file 1: Table S1). These isolates were selected from a culture collection comprising 632 isolates, which were initially identified using sequence variation in the 16S rRNA locus [19]. The 73 isolates came from nine bat species: pallid bat (*Antrozous pallidus*), Townsend’s big-eared bat (*Corynorhinus townsendii*), big brown bat (*Eptesicus fuscus*), silver-haired bat (*Lasionycteris noctivagans*), western small-footed bat (*Myotis ciliolabrum*), long-eared bat (*Myotis evotis*), fringed bat (*Myotis thysanodes*), cave bat (*Myotis velifer*) and long-legged bat (*Myotis volans*). All of these bats are insectivorous, but some have different food preferences depending on when those food items are available. For example, *C. townsendii* prefers to feed on moths compared to beetles [22], whereas the remaining bats in this study consume a variety of hard-bodied arthropods such as beetles. However, when seasonally abundant and energetically worthwhile, these bats will also feed on moths. Herein, the feeding strategies of the bats include aerial hawking or gleaning of arthropods from different surfaces. Of these bats, only *A. pallidus*, *M. evotis* and *M. thysanodes* are considered occasional gleaners and are capable of gleaning insects from plants and other substrates [23–25]. Differences in food preferences and wide foraging ranges provide opportunities for bats to come in contact with a variety of microbes, which may partly explain the genetic variation in *Streptomyces* we observed.

**Fig. 1:**
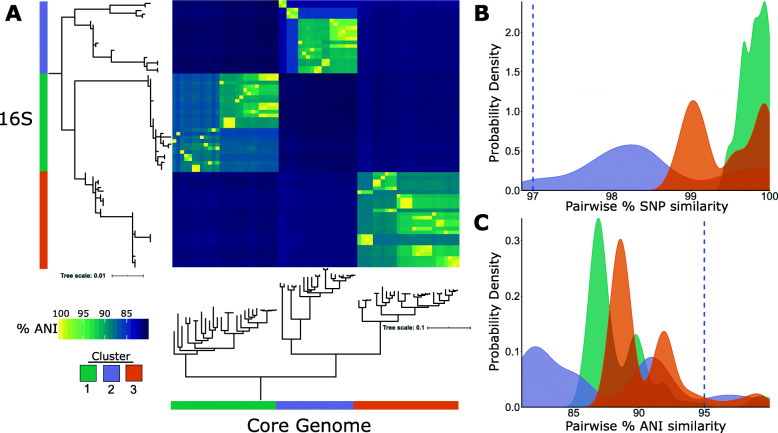
Phylogenetic and genomic comparison of 73 *Streptomyces* strains. **a** Phylogenetic clustering of strains based on 16S rRNA sequence similarity and 1149 core genes. Both trees were midpoint-rooted. Scale bars represent the number of nucleotide substitutions per site. The matrix shows genome-wide, average nucleotide identity (ANI) values calculated for every pair of genomes in the entire data set. Density distribution of all pairwise **b** 16S rRNA sequence similarity and **c** average nucleotide identity (ANI) values. The dashed blue line in each plot indicates the % threshold often used to define a species (97% in 16S rRNA and 95% in ANI). SNP – single nucleotide polymorphisms

The number of contigs per genome ranged from 69 to 500 and N50 values ranged from 31,031–643,063 bp (Additional file 1: Table S1). We initially used sequence variation in the 16S rRNA locus to delineate species boundaries. These isolates can be grouped into three large clusters (Fig. 1a), with each cluster representing a distinct species based on the 97% sequence similarity threshold in the 16S rRNA gene (Fig. 1b and Additional file 2: Fig. S1). This threshold value has been previously used for taxonomic classification of *Streptomyces* [4]. Within each of the three clusters, sequence similarities between isolates ranged from 99.41–99.79%, 96.86–98.47% and 98.76–99.43% in clusters 1, 2 and 3, respectively.

Surprisingly, when we used the genome-wide ANI [21] to compare isolates, the majority of isolates within each of the three 16S rRNA-based clusters fell below the 95% ANI threshold, which is often used to define a species [21] (Fig. 1c and Additional file 3: Table S2). The ANI metric pertains to the average nucleotide identity of all orthologous genes shared between any two genomes and hence provides a more robust comparison in classifying microbial strains [21]. The clustering into three groups that we observed using 16S rRNA sequence variation remained unchanged when we used the 1149 concatenated core genes, which are genes present in ≥95% of the genomes (Additional file 4: Table S3 and Additional file 5: Fig. S2). Within each of the 16S rRNA-defined clusters, genomes exhibited ANI values ranging from 85.53–99.99%, 87.72–99.99%, and 81.03–99.99% in clusters 1, 2 and 3, respectively (Fig. 1a). These results indicate that a species defined by the 16S rRNA gene is made up of multiple species based on their genomic sequences. Overall, we can delineate 41 species of *Streptomyces* using the 95% ANI threshold in our dataset, indicating a greater level of species diversity than was initially recognized.

### Distribution of biosynthetic gene clusters (BGCs)

*Streptomyces* are best known for their prolific ability to produce antibiotics and other useful compounds commonly used in human medicine, animal health, industry, and agriculture [1, 26]. These compounds are derived from the production of secondary metabolites, which are encoded by a set of physically linked genes called BGCs [3, 27]. The genes in a BGC function in peptide assembly, regulation, resistance and synthesis of a secondary metabolite [28, 29]. Secondary metabolites are compounds that are not required for growth but may confer a certain advantage to their producers in a given environment. A previous study of bat-associated *Streptomyces* reported potent antagonistic activity against the fungal pathogen *P. destructans* [19]. Hence, we hypothesized that *Streptomyces* bacteria from southwestern bat species harbor an abundant and diverse suite of BGCs.

**Fig. 2:**
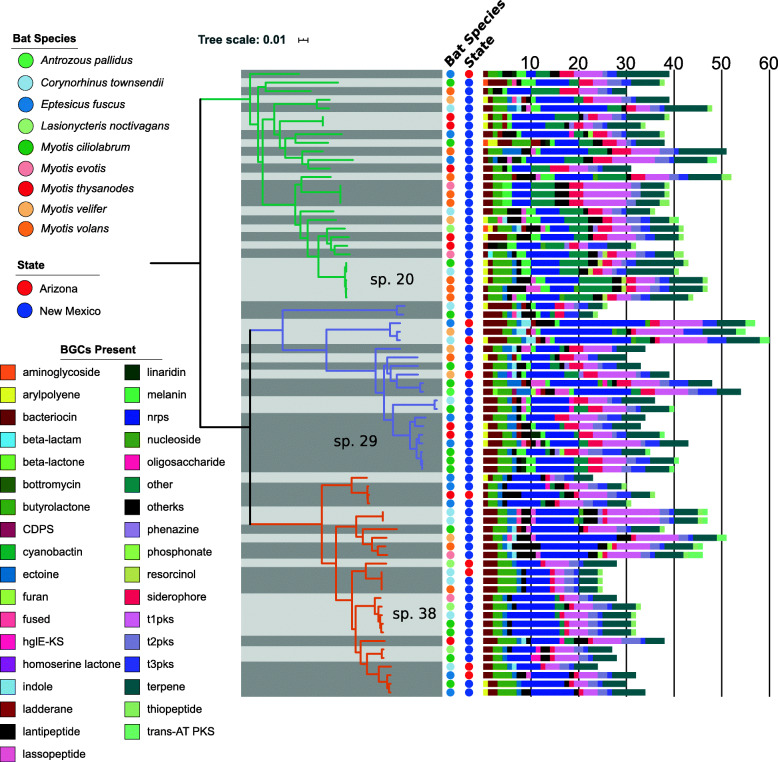
Average nucleotide identity (ANI)-based species boundaries and phylogenetic distribution of biosynthetic gene clusters (BGCs). Species boundaries are delineated using alternating light gray and dark gray boxes and were defined based on the 95% ANI threshold. Three sub-clusters that can be considered as distinct species based on the ANI threshold are tentatively labeled as species 20, 29 and 38. Acronyms: AT – acyltransferase; CDPS – tRNA-dependent cyclodipeptide synthases; hgIE-KS – heterocyst glycolipid synthase-like ketide synthase; NRPS – non-ribosomal peptide synthetase; PKS – polyketide synthase

From our analyses, we detected a total of 32 major classes of BGCs (excluding BGCs identified as others and fused; Fig. 2 and Additional file 6: Table S4), which is consistent with previous studies of *Streptomyces* from other environmental sources [6, 27, 30]. On average, a genome carried 14 distinct classes of BGCs (range = 9–20). Of the 32 BGC classes detected, we found non-ribosomal peptide synthetases (NRPS), siderophores, and terpenes present in all genomes. Other BGCs that were commonly found in *Streptomyces* included bacteriocin (present in 72 genomes), type 1 polyketide synthase (t1PKS; 72 genomes), butyrolactone (71 genomes) and type 3 PKS (t3PKS; 70 genomes). In contrast, some BGCs were present in only a handful of genomes. These included oligosaccharide (8 genomes), linaridin (8 genomes), resorcinol (6 genomes), phosphonate (4 genomes), aminoglycoside (3 genomes), bottromycin (1genome), cyanobactin (1 genome), beta-lactam (1 genome) and homoserine lactone (1 genome). We also note that a genome may harbor multiple copies of a BGC class. For example, the number of NRPS in a single genome ranged from 2 to 18 (mean = 7.5). The number of siderophore copies in a single genome ranged from 1 to 4 (mean = 2.1). The number of terpene copies in a single genome ranged from 3 to 14 (mean = 5.9). We did not identify any specific class of BGC that is unique to any of the three ANI clusters nor to any of the nine bat species.

### Genome variation between strains in three select species

**Fig. 3:**
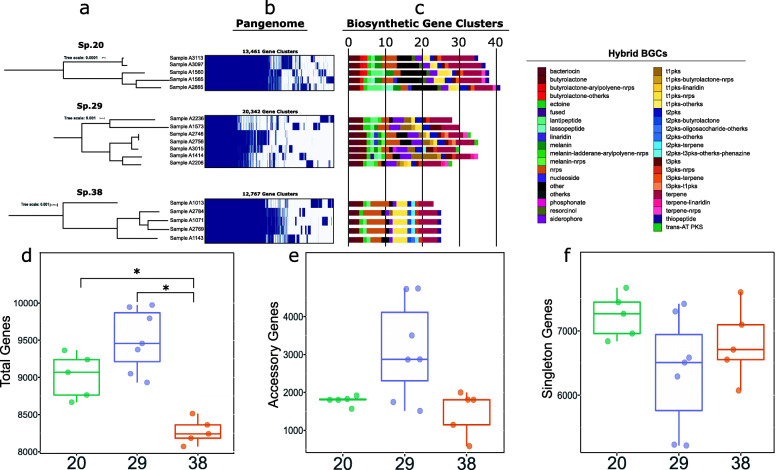
Inter-strain genomic variation in three average nucleotide identity (ANI)-defined species of *Streptomyces* (labeled as species 20, 29 and 38 in Fig. 2). **a** Core genome tree of each species. The number of core genes is 6591, 5125 and 5979 for species 20, 29 and 38, respectively. Scale bars represent the number of nucleotide substitutions per site. **b** Strain distribution of genes in the pangenome of each species. **c** Strain distribution of biosynthetic gene clusters (BGCs) in each species. Hybrid BGCs were classified according to their BGC components. Acronyms: AT – acyltransferase; CDPS – tRNA-dependent cyclodipeptide synthases; hgIE-KS – heterocyst glycolipid synthase-like ketide synthase; NRPS – non-ribosomal peptide synthetase; PKS – polyketide synthase. Comparison of the **d** total number of genes, **e** accessory genes and **f** singleton genes in the pan-genome of each species. * denotes a *p*-value < 0.05. For visual clarity, comparisons that were not statistically significant are not shown. Significance measured using Mann-Whitney U pairwise test. Box plots depict the minimum, first quartile, median, third quartile, and maximum values

We wanted to further investigate the extent of genomic variation among strains within a species. There were three sub-clusters that can be considered as distinct species based on the ANI threshold and that consists of multiple strains. These are tentatively labeled as species 20, 29 and 38 in Fig. 2. These three subclusters each had five, seven and five genomes, respectively (Fig. 3a). We first estimated the pan-genome of each species, which consisted of 13,461, 20,342 and 20,474 genes in species 20, 29 and 38, respectively (Fig. 3b and Additional file 7: Table S5). The total number of core genes were 6591, 5125 and 5979, while the total number of accessory genes were 6870, 15,217 and 14,495 for species 20, 29 and 38, respectively.

We found differences in the number and classes of BGCs, including hybrid BGCs (i.e., BGCs with genes that code for more than one type of scaffold-synthesizing enzymes [28, 31]), between strains of the same species (Fig. 3c). Some major classes of BGCs were present in all genomes across all three species, such as bacteriocin, butyrolactone, ectoine, NRPS, siderophore, types 1, II and III PKS and terpene. However, we also identified BGCs that were strain-specific (lassopeptide, phenazine, and phosphonate in species 29) and species-specific (nucleoside and resorcinol in species 20, and ladderane, linaridin, phenazine, phosphonate and transAT-PKS in species 29). If we further classify the hybrid BGCs based on their individual BGC components, we can further observe greater diversity among strains. For example, we found species-specific cases of butyrolactone-arylpolyene-NRPS (species 20), butyrolactone-other KS (species 20), melanin-ladderane-arylpolyene-NRPS (species 29), melanin-NRPS (species 29), t1PKS-butyrolactone-NRPS (species 29), t1PKS-other KS (species 20), t2PKS-butyrolactone, (species 38) t2PKS-terpene (species 38), t3PKS-t1PKS, (species 29) t3PKS-terpene (species 20), and terpene-linaridin (species 29). We also found strain-specific presence of t1PKS-linaridin, t2PKS-otherKS and t2PKS-t3PKS-otherKS-phenazine.

The total number of protein coding genes per genome ranged from 8666–9364, 8932–9974, 8071–8513 in species 20, 29 and 38, respectively. Species 38 had significantly fewer genes than either species 20 or species 29 (Fig. 3d) (Mann-Whitney U pairwise test). However, we did not find any significant differences in the number of either accessory genes (Fig. 3e) or singleton genes (i.e., genes present only in a single genome) (Fig. 3f) between species. These results indicate that inter-strain variation in gene content further contributes to the overall genetic diversity present in bat-associated *Streptomyces*.

### Recombination within a species

*Streptomyces* are known to frequently recombine [32–34] which may partly explain the observed genomic variation between strains within each of species 20, 29 and 38. Using the core genome alignment of each of the three species, we tested for evidence of recombination using the.

**Fig. 4:**
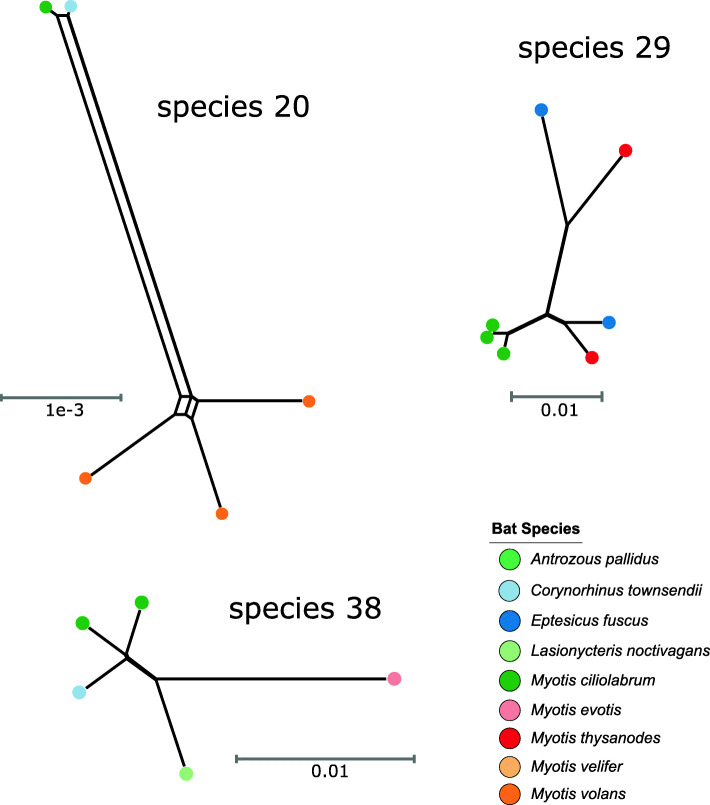
A phylogenetic network of the core genome of *Streptomyces* species 20, 29 and 38 generated using SplitsTree4. The dots represent the host bat species and colors are identical to those in Fig. 2

Pairwise Homoplasy Index test and Splitstree network analysis. The Splitstree analysis shows the reticulations in the phylogenetic relationships between strains of each species (Fig. 4). The networks also reveal that differences in host species do not appear to hinder recombination between strains. However, only species 20 and 38 show significant signal for recombination (*p*-value = 0.0), while species 29 does not (*p*-value = 1.0).

## Discussion

Antibiotic-producing *Streptomyces* bacteria are ubiquitous in nature, yet most studies of its diversity have focused on those free-living strains inhabiting diverse soil environments [5, 6] and those in symbiotic relationship with invertebrates (e.g., insects, marine sponges) [14, 35, 36]. Most commercially used *Streptomyces*-derived antibiotics today, such as streptomycin [37], were originally derived from strains collected from soils. However, investigations on the prevalence, diversity and contributions of *Streptomyces* to their vertebrate animal hosts remain limited. A previous study reported the remarkable species diversity of 20% of a large culture collection of bat-associated *Streptomyces* at the University of New Mexico (UNM) [19]. In that study, sequence variation in the 16S rRNA and five other housekeeping genes were used to characterize the phylogenetic diversity and relationships of *Streptomyces*, with isolates representing 15 novel species that exhibited antifungal activity [19]. Using 73 *Streptomyces* isolates from the UNM culture collection, we uncovered large genomic variation and biosynthetic potential, even among closely related strains. Bats are therefore an important yet under-appreciated source of antibiotic-producing microbes. Our study provides an initial genomic assessment of bat-associated *Streptomyces* that will be critical to prioritizing those strains with the greatest ability to produce novel anti-fungal compounds.

It has long been recognized that species classification in *Streptomyces* is problematic, being driven historically more by the type of antibiotic produced and patent issues rather than by genetic, ecological, or evolutionary data [38]. Recent studies on different *Streptomyces* species defined by the 16S rRNA locus revealed a striking illustration of how traditional species classification can complicate drug discovery schemes or overlook singular bacterial strains. For example, two strains of *Streptomyces griseus* from geographically disparate locations but with identical 16S rRNA sequences, exhibited differences in spore pigmentation, amount of spore formation, aerial hyphae distribution on the colony, and production of secondary metabolites [39]. These differences may be partly explained by adaptive processes to specific environmental conditions. In another study, ten *Streptomyces* strains from different lichen species but with 16S rRNA gene sequences identical to the type strain *Streptomyces cyaneofuscatus* JCM 4364 exhibited highly variable phenotypic (colony morphology and color, halotolerance, optimal pH growth), metabolic and genomic features, such that they could be distinguished as five different species [40].

The large amount of genomic and BGC variation between genomes, even between closely related isolates, may reflect fine-scale differences in their adaptive potential. For example, the presence on all genomes of siderophores, which are involved in the acquisition of ferric ions, may reflect the need for *Streptomyces* to cope in environments with limited iron supply [41]. However, differences in the abundance of siderophores per genome may be due to interactions with specific bacterial partners, as has been previously reported [42, 43]. Future work should focus on investigating the functional role of structurally distinct types of siderophores as well as other BGCs in *Streptomyces*’ adaptation to different bat species that harbor them and to the cave environment.

Our study presents several limitations that need to be recognized. First, only culturable isolates were used in this study. Cultivation techniques, while effective in isolating individuals from a microbial community, can unintentionally bias findings on community composition and functions. This is because cultivation fails to discover novel strains that are recalcitrant to specific cultivation methods, media and laboratory conditions commonly used because they do not precisely mimic the ecological niche of the bacterium in nature. Hence, we recognize that the extent of genomic diversity we found in our dataset is likely an under-estimation of the true scale of *Streptomyces* diversity in bats, suggesting that additional species with unique genetic and phenotypic features remain to be discovered. This will also hold true for exploring BGC diversity. Second, we did not sample *Streptomyces* from the cave walls, where bats may pick up *Streptomyces* spores and hence influence their genetic diversity. Another limitation of our study is the assembly quality of our genomes. Illumina sequencing of large bacterial chromosomes remains a challenge due to chimeric sequences and sequencer errors. Furthermore, genome regions with high G + C content [such as *Streptomyces*, which typically has over 70% G + C content [44]] can be sequenced with lower coverage than the rest of the genome due to biases in the amplification step [45]. These challenges can exacerbate the amount of gene content variation detected in genomes. In this study, the assembly quality of our genomes could take the form of erroneously large accessory genomes and/or improperly characterized BGC content. Future studies should utilize long-read sequencing technologies alongside short-reads to generate more complete hybrid assemblies which can overcome these common sequencing challenges [46].

## Conclusions

Overall, our study provides an initial genome-based assessment of the bat-associated *Streptomyces* diversity that will be critical to prioritizing those strains with the greatest ability to produce novel bioactive compounds, including those that can strongly inhibit *P. destructans* and other mycotic diseases. We emphasize the value of poorly explored settings, such as caves and bats, as important resources of antibiotic-producing bacteria for current drug discovery efforts relevant to human and veterinary medicine.

## Methods

### Collection and isolation of *Streptomyces*

Isolates in our study came from a culture collection of which a subset was used in a previously published dataset of *Streptomyces* sampled from the skin (wing and tail membranes) and fur surfaces of bats [19]. Details on bat collection protocols, sampling permits and bacterial isolation procedures were described in reference [19]. Bats were caught using mist nets or were hand plucked from cave walls, according to approved protocols under the following collection permits: 2014 Arizona and New Mexico Game and Fish Department Scientific Collecting Permit (SP670210, SCI#3423, and SCI#3350), National Park Service Scientific Collecting Permit (CAVE-2014-SCI-0012, CAVE-2015-SCI-0011 ELMA-2013-SCI-0005, ELMA-2014-SCI-0001, CHIR-2015-SCI-0001, and PARA-2012-SCI-0003), U.S. Geological Survey Fort Collins Science Center Standard Operating Procedure (SOP) 2013–01, and an Institutional Animal Care and Use Committee (IACUC) permit from the University of New Mexico (protocol #12–100835-MCC and Protocol 15101307MC) and from the National Park Service (protocol #IMR-ELMA.PARA Northup-Bat-2013.A2 and NPS Protocol Number IMR_ELMA.PARA.CAVE.SEAZ_Northup_Bats_2015.A2). All experimental protocols were approved by the institutions and licensing committees listed above. Bats were swabbed from caves post-hibernation or from netting on the surface near drinking sources. Sampling was carried out in 2013–2015. Four actinobacterium-selective media were used to isolate *Streptomyces* (Actinomycete isolation agar [Difco, Sparks, Maryland], gellan gum agar, humic acid-vitamin agar and glucose yeast extract agar) supplemented with cycloheximide, nalidixic acid, trimethoprim and a vitamin solution. Immediately following swabbing of each bat, plates were inoculated and kept at 4 °C during transport and at 20 °C in the laboratory during initial growth. Initial *Streptomyces* identification was done by extracting and sequencing the 16S rRNA locus using Sanger sequencing [19].

### DNA extraction and whole genome sequencing

Pure cultures were grown in R2B broth medium (Difco, Sparks, Maryland) at 37 °C for 24 –72 h. DNA was extracted and purified from cultures using the DNeasy Extraction kit (Qiagen, Germantown, Maryland) following manufacturer’s protocols. DNA concentration and quality were measured using a Nanodrop spectrophotometer and Qubit 4 fluorometer. DNA libraries were prepared using the NexteraXT protocol (as per the manufacturer’s instructions) with 1 ng of genomic DNA per strain. Samples were sequenced as multiplexed libraries on the Illumina HiSeq platform operated per the manufacturer’s instructions to produce paired end reads of 250 nucleotides in length. Sequencing was done at the University of New Hampshire Hubbard Center for Genome Studies, Durham, New Hampshire, USA.

### Genome assembly and annotation

Reads were assembled into contigs using the de novo assembler SPAdes v.3.13.1 that was developed specifically for bacterial genomes [47]. Genome assembly quality was assessed using QUAST [48]. We also selected only those genomes with < 500 contigs. In total, we used 73 draft genomes for all downstream analyses. The resulting contigs in each genome were annotated using Prokka, a stand-alone tool that combines multiple feature prediction tools to identify coding sequences, ribosomal and transfer RNA genes, non-coding RNA and signal leader peptides in bacterial genomes [49].

### Pan-genome and phylogenetic analysis

To determine the degree of genomic relatedness and hence clarify the taxonomic breadth within our dataset, we calculated the genome-wide ANI for all possible pairs of genomes using the program FastANI v.1.0 [21]. We used the program Roary [50] to characterize the core and accessory genes in the pan-genome of the 73 strains. However, Roary’s default parameters assume a species level relationship among genomes. To account for the greater genomic variation in our genus-scale dataset, we used the mean pairwise fastANI value for the entire genus (81%) as the minimum percent identity between orthologous genes (parameter ‘-i 81). For the species-specific Roary analyses, we used the standard 95% that is commonly used to define species boundaries [50]. Significance in gene content between clusters was measured using Mann-Whitney U pairwise test.

We aligned the sequences of the 16S rRNA gene extracted from the genomes using MAFFT [51] and counted all pairwise single nucleotide polymorphism (SNP) differences using snp-dists v0.6.3 (https://github.com/tseemann/snp-dists). A 16S rRNA phylogenetic tree was built using RAxML v.8.2.11 [52] with a general-time reversible (GTR) nucleotide substitution model [53], four gamma categories for rate heterogeneity and 100 bootstrap replicates. We also built a phylogenetic tree using the concatenated sequence alignments of the core genes using RAxML with the GTR model and four gamma categories. All trees were visualized using the Interactive Tree of Life [54].

BGCs encoding secondary metabolites were predicted and annotated using the standalone version of antiSMASH v.4.1 with default parameters, which identifies BGCs using a signature profile Hidden Markov Model based on multiple sequence alignments of experimentally characterized signature proteins or protein domains [55]. Due to their high number, hybrid BGCs were split and counted as individual BGC classes in the genus tree (e.g., terpene-t1PKS BGC would count as both one terpene and one t1PKS). In species-level comparisons, we showed all unique hybrid BGCs.

### Recombination analysis

Using the alignment of the concatenated core genes of each of the three species as input, we ran the Pairwise Homoplasy Index test for recombination with 100 permutations using PhiPack [56]. The PHI test calculates a pairwise incompatibility score of each nucleotide site in an alignment. The *p*-value for the PHI test was calculated under the null hypothesis of no recombination. Recombinations were visualized using SplitsTree v4.14.4 which integrates reticulations due to recombinations in phylogenetic relationships [57].

All methods were carried out in accordance with relevant guidelines and regulations at the National Park Service, Arizona and New Mexico Game and Fish Departments, U.S. Geological Survey, University of New Mexico and University at Albany.

## Supplementary Information

**Additional file 1: Table S1.** Accession numbers, metadata and genome characteristics of the 73 *Streptomyces* isolates. CDS – coding sequence.

**Additional file 2: Fig. S1.** 16S rRNA tree of *Streptomyces* isolates with bootstrap values.

**Additional file 3: Table S2.** Average nucleotide identity (ANI) values for all pairs of genomes of *Streptomyces* isolates calculated using fastANI.

**Additional file 4: Table S3.** Pan-genome analysis of the 73 *Streptomyces* genomes estimated using Roary.

**Additional file 5: Fig. S2.** Core genome tree generated from 1149 concatenated core genes of *Streptomyces* identified by Roary.

**Additional file 6: Table S4.** Biosynthetic gene clusters (BGCs) of all genomes of *Streptomyces* estimated using antiSMASH.

**Additional file 7: Table S5.** Pan-genome analyses of *Streptomyces* sp. 20, 29 and 38 in Fig. 2 estimated using Roary.

## Data Availability

Genome sequence data of the 73 isolates have been deposited in the NCBI Sequence Read Archive under BioProject ID PRJNA673820 with BioSample accession numbers listed in Additional file 1: Table S1. The raw data in the NCBI database serve as an acceptable repository by U.S. Geological Survey’s standards.

## References

[1] Procópio RE d L, Silva IR d, Martins MK, Azevedo JL d, Araújo JM d (2012). Antibiotics produced by Streptomyces. Braz J Infect Dis.

[2] van der Meij A, Worsley SF, Hutchings MI, van Wezel GP (2017). Chemical ecology of antibiotic production by actinomycetes. FEMS Microbiol Rev.

[3] van Bergeijk DA, Terlouw BR, Medema MH, van Wezel GP (2020). Ecology and genomics of Actinobacteria: new concepts for natural product discovery. Nat Rev Microbiol.

[4] Labeda DP, Goodfellow M, Brown R, Ward AC, Lanoot B, Vanncanneyt M, Swings J, Kim SB, Liu Z, Chun J, Tamura T, Oguchi A, Kikuchi T, Kikuchi H, Nishii T, Tsuji K, Yamaguchi Y, Tase A, Takahashi M, Sakane T, Suzuki KI, Hatano K (2012). Phylogenetic study of the species within the family Streptomycetaceae. Antonie Van Leeuwenhoek.

[5] Laskaris P, Tolba S, Calvo-Bado L, Wellington EM, Wellington L (2010). Coevolution of antibiotic production and counter-resistance in soil bacteria. Environ Microbiol.

[6] Nicault M, Tidjani A-R, Gauthier A, Dumarcay S, Gelhaye E, Bontemps C (2020). Mining the biosynthetic potential for specialized metabolism of a Streptomyces soil community. Antibiotics (Basel).

[7] Augustine N, Wilson PA, Kerkar S, Thomas S (2012). Arctic actinomycetes as potential inhibitors of Vibrio cholerae biofilm. Curr Microbiol.

[8] Encheva-Malinova M, Stoyanova M, Avramova H, Pavlova Y, Gocheva B, Ivanova I, Moncheva P (2014). Antibacterial potential of streptomycete strains from Antarctic soils. Biotechnol Biotechnol Equip.

[9] Arocha-Garza HF, Canales-Del Castillo R, Eguiarte LE, Souza V, De la Torre-Zavala S (2017). High diversity and suggested endemicity of culturable Actinobacteria in an extremely oligotrophic desert oasis. PeerJ.

[10] Zhao F, Qin Y-H, Zheng X, Zhao H-W, Chai D-Y, Li W, Pu MX, Zuo XS, Qian W, Ni P, Zhang Y, Mei H, He ST (2016). Biogeography and adaptive evolution of Streptomyces strains from saline environments. Sci Rep.

[11] Sarmiento-Vizcaíno A, González V, Braña AF, Palacios JJ, Otero L, Fernández J, Molina A, Kulik A, Vázquez F, Acuña JL, García LA, Blanco G (2017). Pharmacological potential of phylogenetically diverse actinobacteria isolated from Deep-Sea coral ecosystems of the submarine Avilés canyon in the Cantabrian Sea. Microb Ecol.

[12] Seipke RF, Kaltenpoth M, Hutchings MI (2012). Streptomyces as symbionts: an emerging and widespread theme?. FEMS Microbiol Rev.

[13] Kaltenpoth M, Göttler W, Herzner G, Strohm E (2005). Symbiotic bacteria protect wasp larvae from fungal infestation. Curr Biol.

[14] Kroiss J, Kaltenpoth M, Schneider B, Schwinger M-G, Hertweck C, Maddula RK, Strohm E, Svatoš A (2010). Symbiotic Streptomycetes provide antibiotic combination prophylaxis for wasp offspring. Nat Chem Biol.

[15] Riquelme C, Marshall Hathaway JJ, Enes Dapkevicius M d LN, Miller AZ, Kooser A, Northup DE (2015). Actinobacterial diversity in volcanic caves and associated geomicrobiological interactions. Front Microbiol.

[16] Axenov-Gribanov DV, Axenov-Gibanov DV, Voytsekhovskaya IV, Tokovenko BT, Protasov ES, Gamaiunov SV (2016). Actinobacteria isolated from an underground lake and moonmilk speleothem from the biggest conglomeratic karstic cave in Siberia as sources of novel biologically active compounds. PLoS One.

[17] Adam D, Maciejewska M, Naômé A, Martinet L, Coppieters W, Karim L (2018). Isolation, characterization, and antibacterial activity of hard-to-culture Actinobacteria from cave moonmilk deposits. Antibiotics (Basel).

[18] Wiseschart A, Mhuantong W, Tangphatsornruang S, Chantasingh D, Pootanakit K (2019). Shotgun metagenomic sequencing from Manao-Pee cave, Thailand, reveals insight into the microbial community structure and its metabolic potential. BMC Microbiol.

[19] Hamm PS, Caimi NA, Northup DE, Valdez EW, Buecher DC, Dunlap CA, et al. Western bats as a reservoir of novel Streptomyces species with antifungal activity. Appl Environ Microbiol. 2017;83(5). 10.1128/AEM.03057-16. 10.1128/AEM.03057-16 PMC5311414 27986729

[20] Drees KP, Lorch JM, Puechmaille SJ, Parise KL, Wibbelt G, Hoyt JR, et al. Phylogenetics of a fungal invasion: origins and widespread dispersal of white-nose syndrome. mBio. 2017;8(6):e01941–17. 10.1128/mBio.01941-17. 10.1128/mBio.01941-17 PMC5727414 29233897

[21] Jain C, Rodriguez-R LM, Phillippy AM, Konstantinidis KT, Aluru S (2018). High throughput ANI analysis of 90K prokaryotic genomes reveals clear species boundaries. Nat Commun.

[22] Kunz TH, Martin RA (1982). Plecotus townsendii. Mamm Species.

[23] O’Farrell MJ, Studier EH (1980). *Myotis thysanodes*. Mammalian species.

[24] Manning RW, Jones JK (1989). Myotis evotis. Mamm Species.

[25] Ammerman LK, Hice CL, Schmidley DJ (2012). Bats of Texas.

[26] Bĕhal V (2000). Bioactive products from Streptomyces. Adv Appl Microbiol.

[27] Doroghazi JR, Metcalf WW (2013). Comparative genomics of actinomycetes with a focus on natural product biosynthetic genes. BMC Genomics.

[28] Cimermancic P, Medema MH, Claesen J, Kurita K, Wieland Brown LC, Mavrommatis K, Pati A, Godfrey PA, Koehrsen M, Clardy J, Birren BW, Takano E, Sali A, Linington RG, Fischbach MA (2014). Insights into secondary metabolism from a global analysis of prokaryotic biosynthetic gene clusters. Cell.

[29] Medema MH, Kottmann R, Yilmaz P, Cummings M, Biggins JB, Blin K, de Bruijn I, Chooi YH, Claesen J, Coates RC, Cruz-Morales P, Duddela S, Düsterhus S, Edwards DJ, Fewer DP, Garg N, Geiger C, Gomez-Escribano JP, Greule A, Hadjithomas M, Haines AS, Helfrich EJN, Hillwig ML, Ishida K, Jones AC, Jones CS, Jungmann K, Kegler C, Kim HU, Kötter P, Krug D, Masschelein J, Melnik AV, Mantovani SM, Monroe EA, Moore M, Moss N, Nützmann HW, Pan G, Pati A, Petras D, Reen FJ, Rosconi F, Rui Z, Tian Z, Tobias NJ, Tsunematsu Y, Wiemann P, Wyckoff E, Yan X, Yim G, Yu F, Xie Y, Aigle B, Apel AK, Balibar CJ, Balskus EP, Barona-Gómez F, Bechthold A, Bode HB, Borriss R, Brady SF, Brakhage AA, Caffrey P, Cheng YQ, Clardy J, Cox RJ, de Mot R, Donadio S, Donia MS, van der Donk WA, Dorrestein PC, Doyle S, Driessen AJM, Ehling-Schulz M, Entian KD, Fischbach MA, Gerwick L, Gerwick WH, Gross H, Gust B, Hertweck C, Höfte M, Jensen SE, Ju J, Katz L, Kaysser L, Klassen JL, Keller NP, Kormanec J, Kuipers OP, Kuzuyama T, Kyrpides NC, Kwon HJ, Lautru S, Lavigne R, Lee CY, Linquan B, Liu X, Liu W, Luzhetskyy A, Mahmud T, Mast Y, Méndez C, Metsä-Ketelä M, Micklefield J, Mitchell DA, Moore BS, Moreira LM, Müller R, Neilan BA, Nett M, Nielsen J, O'Gara F, Oikawa H, Osbourn A, Osburne MS, Ostash B, Payne SM, Pernodet JL, Petricek M, Piel J, Ploux O, Raaijmakers JM, Salas JA, Schmitt EK, Scott B, Seipke RF, Shen B, Sherman DH, Sivonen K, Smanski MJ, Sosio M, Stegmann E, Süssmuth RD, Tahlan K, Thomas CM, Tang Y, Truman AW, Viaud M, Walton JD, Walsh CT, Weber T, van Wezel GP, Wilkinson B, Willey JM, Wohlleben W, Wright GD, Ziemert N, Zhang C, Zotchev SB, Breitling R, Takano E, Glöckner FO (2015). Minimum information about a biosynthetic gene cluster. Nat Chem Biol.

[30] Belknap KC, Park CJ, Barth BM, Andam CP (2020). Genome mining of biosynthetic and chemotherapeutic gene clusters in Streptomyces bacteria. Sci Rep.

[31] Zotchev SB, Pontarotti P (2014). Genomics-based insights into the evolution of secondary metabolite biosynthesis in actinomycete bacteria. Evolutionary biology: genome evolution, speciation, coevolution and origin of life.

[32] Doroghazi JR, Buckley DH (2010). Widespread homologous recombination within and between Streptomyces species. ISME J.

[33] Park CJ, Andam CP (2019). Within-species genomic variation and variable patterns of recombination in the tetracycline producer Streptomyces rimosus. Front Microbiol.

[34] Tidjani A-R, Lorenzi J-N, Toussaint M, van Dijk E, Naquin D, Lespinet O, et al. Massive gene flux drives genome diversity between sympatric Streptomyces conspecifics. mBio. 2019;10(5):e01533–19. 10.1128/mBio.01533-19. 10.1128/mBio.01533-19 PMC6722414 31481382

[35] Scott JJ, Oh D-C, Yuceer MC, Klepzig KD, Clardy J, Currie CR (2008). Bacterial protection of beetle-fungus mutualism. Science.

[36] Almeida EL, Carrillo Rincón AF, Jackson SA, Dobson ADW (2019). Comparative genomics of marine sponge-derived Streptomyces spp. isolates SM17 and SM18 with their closest terrestrial relatives provides novel insights into environmental niche adaptations and secondary metabolite biosynthesis potential. Front Microbiol.

[37] Waksman SA (1953). Streptomycin: background, isolation, properties, and utilization. Science.

[38] Anderson AS, Wellington EM (2001). The taxonomy of Streptomyces and related genera. Int J Syst Evol Microbiol.

[39] Sottorff I, Wiese J, Lipfert M, Preußke N, Sönnichsen FD, Imhoff JF. Different secondary metabolite profiles of phylogenetically almost identical Streptomyces griseus strains originating from geographically remote locations. Microorganisms. 2019;7(6). 10.3390/microorganisms7060166. 10.3390/microorganisms7060166 PMC6616549 31174336

[40] Antony-Babu S, Stien D, Eparvier V, Parrot D, Tomasi S, Suzuki MT (2017). Multiple Streptomyces species with distinct secondary metabolomes have identical 16S rRNA gene sequences. Sci Rep.

[41] Eto D, Watanabe K, Saeki H, Oinuma K, Otani K, Nobukuni M, Shiratori-Takano H, Takano H, Beppu T, Ueda K (2013). Divergent effects of desferrioxamine on bacterial growth and characteristics. J Antibiot (Tokyo).

[42] Traxler MF, Watrous JD, Alexandrov T, Dorrestein PC, Kolter R (2013). Interspecies interactions stimulate diversification of the Streptomyces coelicolor secreted metabolome. mBio.

[43] Kramer J, Özkaya Ö, Kümmerli R (2020). Bacterial siderophores in community and host interactions. Nat Rev Microbiol.

[44] Barka EA, Vatsa P, Sanchez L, Gaveau-Vaillant N, Jacquard C, Klenk H-P (2015). Taxonomy, physiology, and natural products of Actinobacteria. Microbiol Mol Biol Rev.

[45] Aird D, Ross MG, Chen W-S, Danielsson M, Fennell T, Russ C, Jaffe DB, Nusbaum C, Gnirke A (2011). Analyzing and minimizing PCR amplification bias in Illumina sequencing libraries. Genome Biol.

[46] Lee N, Kim W, Hwang S, Lee Y, Cho S, Palsson B, Cho BK (2020). Thirty complete Streptomyces genome sequences for mining novel secondary metabolite biosynthetic gene clusters. Sci Data.

[47] Bankevich A, Nurk S, Antipov D, Gurevich AA, Dvorkin M, Kulikov AS, Lesin VM, Nikolenko SI, Pham S, Prjibelski AD, Pyshkin AV, Sirotkin AV, Vyahhi N, Tesler G, Alekseyev MA, Pevzner PA (2012). SPAdes: a new genome assembly algorithm and its applications to single-cell sequencing. J Comput Biol.

[48] Gurevich A, Saveliev V, Vyahhi N, Tesler G (2013). QUAST: quality assessment tool for genome assemblies. Bioinformatics.

[49] Seemann T (2014). Prokka: rapid prokaryotic genome annotation. Bioinformatics.

[50] Page AJ, Cummins CA, Hunt M, Wong VK, Reuter S, Holden MTG, Fookes M, Falush D, Keane JA, Parkhill J (2015). Roary: rapid large-scale prokaryote pan genome analysis. Bioinformatics.

[51] Katoh K, Standley DM (2013). MAFFT multiple sequence alignment software version 7: improvements in performance and usability. Mol Biol Evol.

[52] Stamatakis A (2014). RAxML version 8: a tool for phylogenetic analysis and post-analysis of large phylogenies. Bioinformatics.

[53] Tavaré S (1986). Some probabilistic and statistical problems in the analysis of DNA sequences. American mathematical society: lectures on mathematics in the life sciences.

[54] Letunic I, Bork P (2016). Interactive tree of life (iTOL) v3: an online tool for the display and annotation of phylogenetic and other trees. Nucleic Acids Res.

[55] Blin K, Wolf T, Chevrette MG, Lu X, Schwalen CJ, Kautsar SA, Suarez Duran HG, de los Santos ELC, Kim HU, Nave M, Dickschat JS, Mitchell DA, Shelest E, Breitling R, Takano E, Lee SY, Weber T, Medema MH (2017). antiSMASH 4.0-improvements in chemistry prediction and gene cluster boundary identification. Nucleic Acids Res.

[56] Bruen TC, Philippe H, Bryant D (2006). A simple and robust statistical test for detecting the presence of recombination. Genetics.

[57] Huson DH (1998). SplitsTree: analyzing and visualizing evolutionary data. Bioinformatics.

