# Supplementary material for: Unexpected genomic, biosynthetic and species diversity of Streptomyces bacteria from bats in Arizona and New Mexico, USA
Source: BMC Genomics. 2021 Apr 7;22:247. doi: 10.1186/s12864-021-07546-w (PMC8028829; doi:10.1186/s12864-021-07546-w)
Supplement: Supplementary file 2 — Additional file 2: Fig. S1. 16S rRNA tree of Streptomyces isolates with bootstrap values. [file 12864_2021_7546_MOESM2_ESM.pdf]

Supplementary Material for:

Unexpected genomic, biosynthetic and species diversity of *Streptomyces* bacteria from bats in Arizona and New Mexico, USA

Cooper J. Park[a], Nicole A. Caimi[b], Debbie C. Buecher[c], Ernest W. Valdez[b,d], Diana E. Northup[b*], Cheryl P. Andam[e*]

[a] Department of Molecular, Cellular and Biomedical Sciences, University of New Hampshire, Durham, New Hampshire, USA
[b] Department of Biology, University of New Mexico, Albuquerque, New Mexico, USA
[c] Buecher Biological Consulting, Tucson, Arizona, USA
[d] U.S. Geological Survey, Fort Collins Science Center, Fort Collins, Colorado, USA
[e] Department of Biological Sciences, University at Albany, State University of New York, Albany, New York, USA

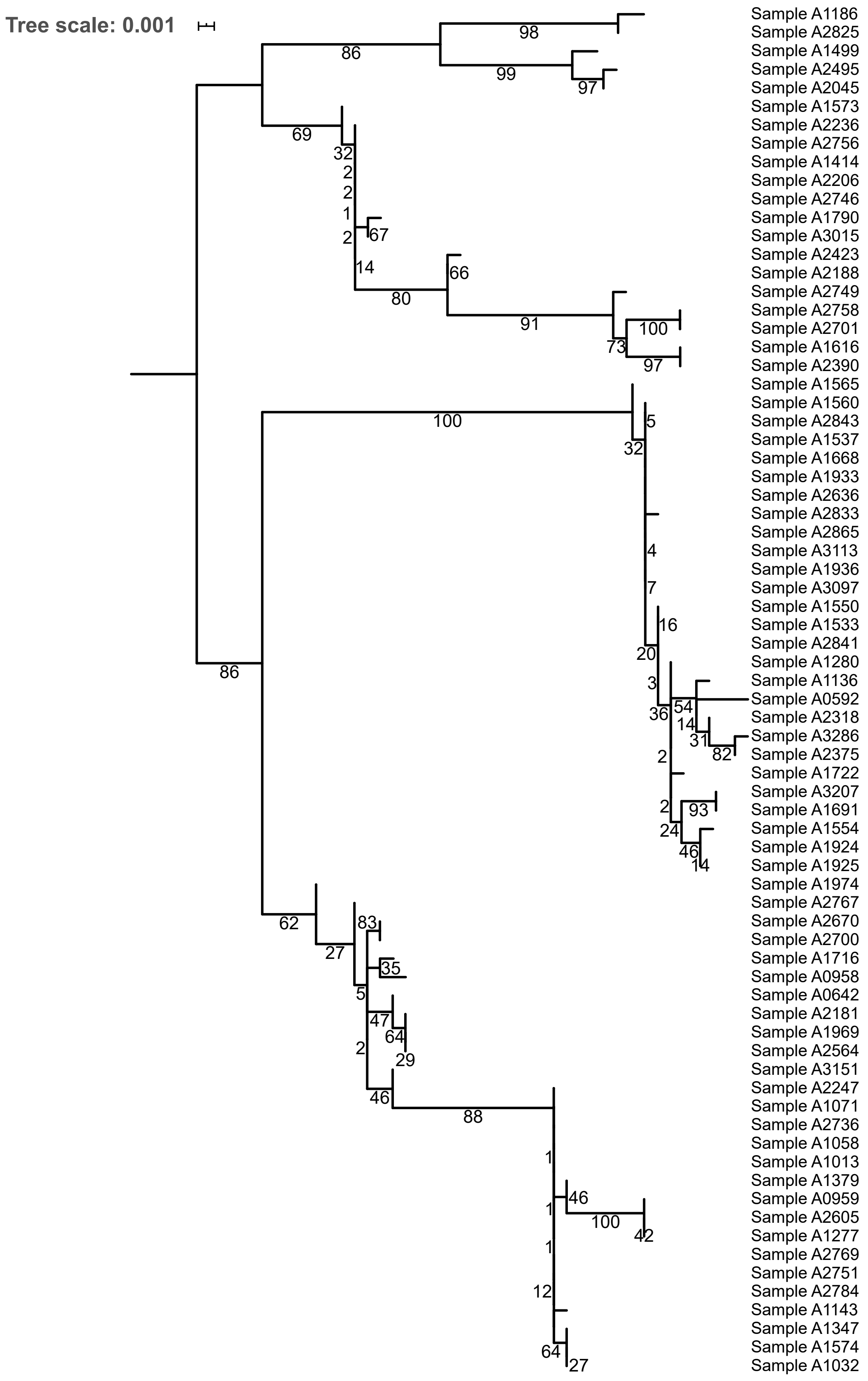

Supplementary Figure S1. 16S rRNA tree with bootstrap values
