# Supplementary material for: Unexpected genomic, biosynthetic and species diversity of Streptomyces bacteria from bats in Arizona and New Mexico, USA
Source: BMC Genomics. 2021 Apr 7;22:247. doi: 10.1186/s12864-021-07546-w (PMC8028829; doi:10.1186/s12864-021-07546-w)
Supplement: Supplementary file 5 — Additional file 5: Fig. S2. Core genome tree generated from 1149 concatenated core genes of Streptomyces identified by Roary. [file 12864_2021_7546_MOESM5_ESM.pdf]

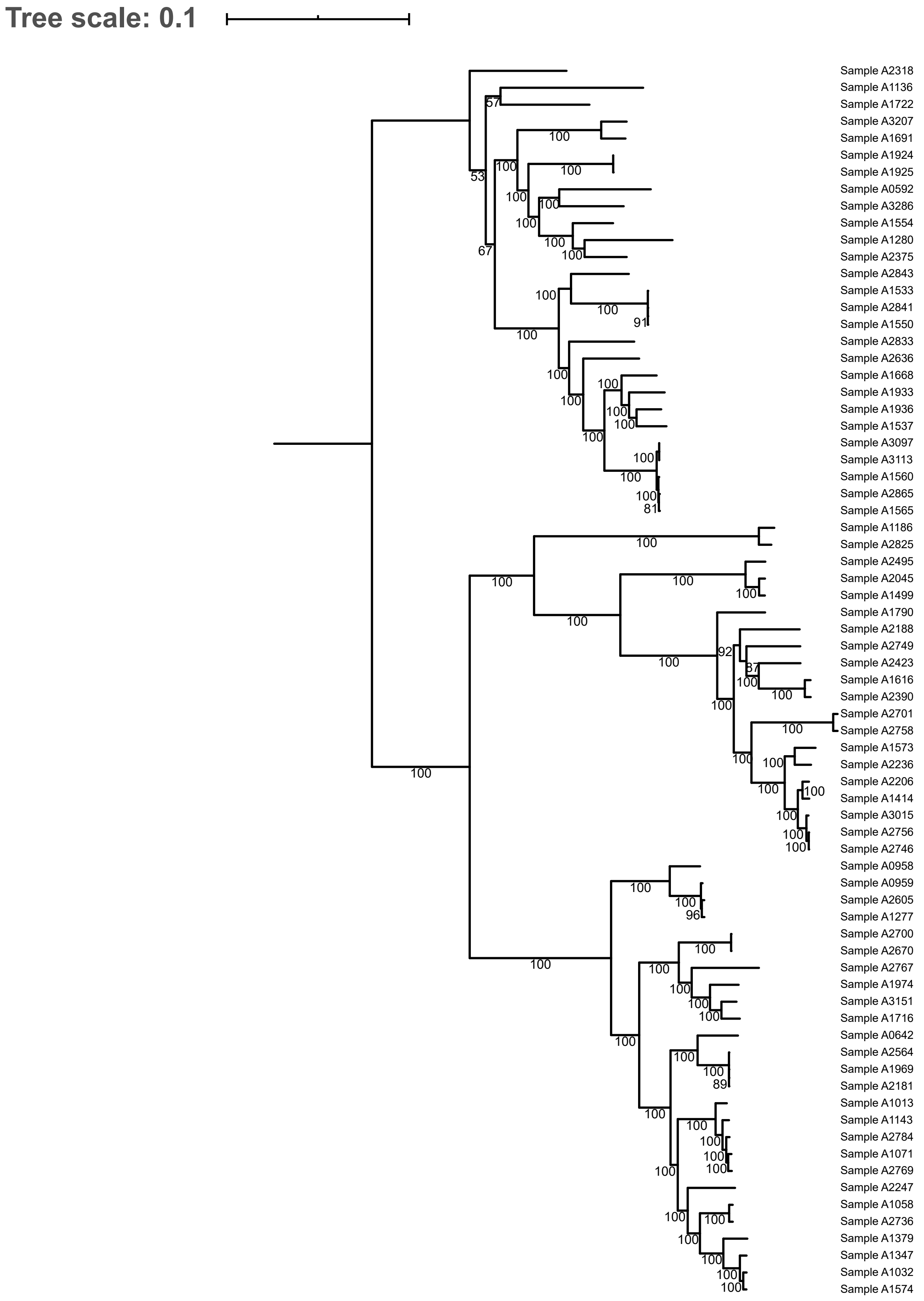

Supplementary Figure S2. Core genome tree generated from 1,149 concatenated core genes identified by Roary
